# Second-generation drug-eluting stents in the elderly patients with acute coronary syndrome: the in-hospital and 12-month follow-up of the all-comer registry

**DOI:** 10.1007/s40520-016-0649-8

**Published:** 2016-11-10

**Authors:** Wojciech Wańha, Damian Kawecki, Tomasz Roleder, Beata Morawiec, Sylwia Gładysz, Adam Kowalówka, Tomasz Jadczyk, Barbara Adamus, Tomasz Pawłowski, Grzegorz Smolka, Maciej Kaźmierski, Andrzej Ochała, Ewa Nowalany-Kozielska, Wojciech Wojakowski

**Affiliations:** 1 Third Division of Cardiology, Medical University of Silesia, Katowice, Poland; 2 Second Division of Cardiology, Medical University of Silesia, Zabrze, Poland; 3 Division of Cardiothoracic Surgery, Medical University of Silesia, Katowice, Poland; 4 Division of Cardiology, Specialist Hospital, Wilkowice, Poland

**Keywords:** Percutaneous coronary intervention, Drug-eluting stents, Elderly patients

## Abstract

**Background:**

Katowice–Zabrze registry provides data that can be used to evaluate clinical outcomes of percutaneous coronary interventions in elderly patients (≥70 y/o) treated with either first- (DES-I) or second-generation (DES-II) drug-eluting stents (DES).

**Methods:**

The registry consisted of data from 1916 patients treated with coronary interventions using either DES-I or DES-II stents. For our study, we defined patients ≥70 years of age as elderly. We evaluated any major adverse cardiac and cerebral events (MACCE) at 12-month follow-up.

**Results:**

Coronary angiography revealed a higher incidence of multivessel coronary artery disease in this elderly patient population. There were no differences in acute and subacute stent thrombosis (0.4 vs. 0.6%,* p *= 0.760; 0.4 vs. 0.4%; *p* = 0.712). Elderly patients experienced more in-hospital bleeding complications requiring blood transfusion (2.0 vs. 0.9%; *p* = 0.003). Resuscitated cardiac arrests (2.0 vs. 0.9%; *p* = 0.084) were observed more often in this elderly patients during hospitalization. The composite in-hospital MACCE rates did not differ statistically between both groups (1.4 vs. 1.1%; *p* = 0.567). Data from a twelve-month follow-up disclosed that mortality was higher (7.1 vs. 1.8%; *p* < 0.001) in the elderly, with no difference in TVR (7.2 vs. 9.9%, *p* = 0.075), MI (6.0 vs. 4.8%, *p* = 0.300), stroke (0.8 vs. 0.6%, *p* = 0.600) and composite MACCE (15.0 vs. 13.4%, *p* = 0.324). The age of 70 years or over was an independent predictor of death [HR = 2.55 (95% CI 1.49–4.37); *p* < 0.001]. The use of DES-II reduced the risk of MI [HR = 0.40 (95% CI 0.19–0.82); *p* = 0.012] in the elderly.

**Conclusion:**

This elderly patient population had an increased risk of in-hospital bleeding complications requiring blood transfusion and a higher risk of death at 12-month follow-up. The use of new-generation DES reduced the risk of MI in the elderly population.

## Background

Elderly patients represent an increasing percentage of the population undergoing percutaneous coronary interventions (PCI) [1]. This trend is due, in part, to a prolonged life expectancy and better access to medical care. However, advanced age is associated with poor outcomes in patients with acute coronary syndromes (ACS), stable coronary artery disease (CAD) treated with PCI and bypass surgery as well as in patients with heart failure [2–4]. Elderly patients undergoing PCI have a significantly higher burden of comorbidities.

When compared with younger patients, they less often receive guideline-recommended adjunctive therapies [2], and invasive treatment with drug-eluting stents (DES) implantation [5, 6]. Moreover, these patients are often excluded from randomized clinical trials which perhaps could elucidate optimal therapies and risk assessments that would reduce complication specific to this population [7, 8]. Furthermore, there are limited data on the impact of the DES types (first vs. second generation: DES-I vs. DES-II) on PCI outcomes in the elderly. The primary goal of this present study was to evaluate the effect of age on the risk of major adverse cardiovascular events at 12-month follow-up in patients treated with DES. The secondary goal was to compare the impact of DES types on the observed outcome in these elderly patients.

## Methods and study population

The Katowice–Zabrze registry contains data that included 1916 patients treated with either first- (paclitaxel and sirolimus eluting; 33.6%) or second-generation (everolimus, zotarolimus, biolimus A9 eluting, 66.4%) DES in two tertiary Silesian cardiology centers. We obtained and recorded retrospective data which included baseline characteristics, cardiac history, risk factors, medications, angiographic and procedural data. For our study, we defined patients ≥70 years of age as elderly. Angiographic data were collected in all patients undergoing PCI and recorded in the cardiovascular information registry. SYNTAX scores were calculated for all patients except these with prior CABG (coronary artery bypass graft). For patients with the occluded infarct-related artery, SYNTAX scores were calculated based on baseline angiography. Two observers estimated the SYNTAX scores, in cases where the SYNTAX score consensus could not be made; the angiography was excluded from this analysis. The primary efficacy endpoint was a composite of major adverse cardiac and cerebral events (MACCE), including all-cause death, non-fatal myocardial infarction (MI), target vessel revascularization (TVR), and stroke during the in-hospital stay and at 12-month follow-up. The secondary endpoints were individual components of the primary endpoint (all-cause death, MI, TVR, stroke) and in-hospital bleeding complications. The safety of DES was defined as definite stent thrombosis (acute, subacute, late). TVR, definite stent thrombosis, acute, subacute and late stent thrombosis were defined according to the definitions of endpoints for clinical trials [9]. Gastrointestinal bleeding was considered an endpoint if it fulfilled criteria for type 3 or type 5 bleeding complication according to proposed definitions [10]. Data regarding outcomes (MACCE and gastrointestinal bleeding) at 12-month were obtained from the database of the National Health Fund Service (Ministry of Health).

## Statistics

Statistical analysis was performed using MedCalc Software (v.12 Belgium). Continuous data were presented as mean ± standard deviation and median with interquartile range (Q1–Q3). Qualitative data were expressed as crude values and/or percentages. Between-group differences for quantitative variables were assessed using Mann–Whitney U test for non-normally distributed data and one-way ANOVA for normally distributed data. Chi-square test was used for qualitative variables. Data distribution was verified with Smirnov–Kolmogorov test. The univariable analysis was performed to determine the risk factors for death at 12-month follow-up. Variables with *p* values less than 0.05 entered the multivariate analysis model to estimate independent risk factors of death at 12-month follow-up. Kaplan–Meier curves were used to present the unadjusted time-to-event data for investigated end-points. A value of 2-tailed *p* < 0.05 was considered significant.

## Results

The registry included 1916 patients referred for PCI because of unstable angina (UA) [1500 (78.2%)], non-ST-segment elevation myocardial infarction (NSTEMI) [285 (14.8%)] and ST-segment elevation myocardial infarction (STEMI/LBBB) [131(6.8%)] (Table 1). Five hundred sixty-three patients (29.4%) were ≥70 years of age compared to one thousand, three hundred and fifty-three patients (70.6%) ≤70 years of age. There were fewer men in the elderly group, and elderly patients had a higher prevalence of hypertension, diabetes, chronic kidney disease, anemia, chronic obstructive pulmonary disease, carotid artery disease and neoplasm, as compared to the younger group. The elderly patients were more often hospitalized because of NSTEMI and had a higher prevalence of GRACE risk score over 140. The length of hospital stay was also longer in elderly patients (5.0 IQR 4–7 vs. 4.0 IQR 3–6, *p* < 0.001). Younger patients had more dyslipidemia, family history of CAD, and were more often current smokers. There were no differences regarding the history of myocardial infarction, previous PCI and CABG between the elderly and younger patients’ groups. Elderly patients had a lower LVEF (50% IQR 44.0–58.0 vs. 55% IQR 46.0–60.0; *p* = 0.001) when compared to the younger group (Table 1).

**Table 1:** Patients characteristics, risk factors and clinical presentation according to the age

	Age <70 *n* = 1353 (70.6%)	Age ≥70 *n* = 563 (29.4%)	*p*
Demographic data			
Male, *n* (%)	944 (69.7)	292 (51.8)	<0.001
BMI (kg/m^2^), median (IQR)	28.7 (25.8–31.6)	28.4 (25.5–31.4)	0.623
Discharge diagnosis			
UA, *n* (%)	1074 (79.4)	426 (75.6)	0.082
NSTEMI, *n* (%)	178 (13.2)	107 (19.0)	0.001
STEMI/LBBB, *n* (%)	101 (7.5)	30 (5.3)	0.112
CAD history			
Previous MI, *n* (%)	638 (47.2)	277 (49.2)	0.443
Previous PCI, *n* (%)	744 (55.0)	320 (56.8)	0.489
Previous CABG, *n* (%)	277 (20.5)	121 (21.4)	0.660
CAD risk factors			
Hypertension, *n* (%)	1137 (84.0)	506 (89.9)	0.001
Dyslipidemia, *n* (%)	946 (69.9)	318 (56.5)	<0.001
CKD, *n* (%)	141 (10.4)	190 (33.7)	<0.001
Anemia, *n* (%)	113 (8.4)	104 (18.5)	<0.001
Diabetes mellitus, *n* (%)	440 (32.5)	277 (49.2)	<0.001
Current smoking, *n* (%)	418 (30.9)	46 (8.2)	<0.001
Family history of CAD, *n* (%)	501 (37.0)	127 (22.6)	<0.001
Concomitant disease			
Cancer, *n* (%)	63 (4.7)	54 (9.6)	<0.001
COPD, *n* (%)	64 (4.7)	53 ( (9.4)	<0.001
PAD, *n* (%)	147 (10.9)	71 (12.6)	0.308
Carotid artery disease, *n* (%)	64 (4.7)	49 (8.7)	0.002
Obesity, *n* (%)	319 (23.6)	122 (21.7)	0.398
Length of hospital stay (day), median (IQR)	4.0 (3–6)	5.0 (4–7)	<0.001
Left ventricular function, *n* (%)			
<30%	81 (6.0)	32 (5.7)	0.890
30–50%	292 (21.6)	162 (28.8)	<0.001
>50%	964 (71.2)	361 (64.1)	0.002
LVEF, median (IQR)	55.0 (46.0–60.0)	50.0 (44.0–58.0)	0.001
Laboratory (on admission)			
GFR (ml/min/1.73 m^2^), median (IQR)	88.2 (73.5–97.3)	67.7 (55.1–82.3)	<0.001
Hemoglobin, (g/dl), median (IQR)	14.4 (13.5–15.2)	13.6 (12.7–14.5)	<0.001
Clinical status on admission			
HR, (bpm), median (IQR)	70 (60–80)	70 (60–76)	0.935
SBP, (mmHg), median (IQR)	130 (120–145)	140 (125–150)	<0.001
GRACE score > 140, *n* (%)	84 (6.2)	52 (9.2)	0.007

CKD was defined as estimated GFR (eGFR) <60 60 ml/min/1.73 m^2^ calculated using the modification of diet in renal disease (MDRD) method

*BMI* body mass index, *UA* unstable angina, *NSTEMI* non-ST-segment elevation myocardial infarction, *STEMI* ST-segment elevation myocardial infarction, *CAD* coronary artery disease, *MI* myocardial infarction, *PCI* percutaneous coronary intervention, *CABG* coronary artery bypass graft, *CKD* chronic kidney disease, *COPD* chronic obstructive pulmonary disease, *PAD* peripheral artery disease, *HR* heart rate, *SBP* systolic blood pressure

### Chronic medications

The higher burden of comorbidities observed in the elderly patient population included atrial fibrillation. Therefore, the use of vitamin K antagonists at discharge was higher in the elderly group (Table 2).

**Table 2:** Post-procedure drug therapy according to the age

	Age <70 *n* = 1353 (70.6%)	Age ≥70 *n* = 563 (29.4%)	*p*
ASA, *n* (%)	1335 (98.7)	552 (98.0)	0.270
Clopidogrel, *n* (%)	1334 (98.6)	555 (98.6)	0.988
VKA, *n* (%)	46 (3.3)	47 (8.3)	<0.001
Beta-blockers, *n* (%)	1220 (90.2)	498 (88.5)	0.261
ACEI, *n* (%)	1073 (79.3)	442 (78.5)	0.706
ARB, *n* (%)	157 (11.6)	73 (13.0)	0.453
Statins, *n* (%)	1260 (93.1)	530 (94.1)	0.524
Ca-blockers, *n* (%)	337 (24.9)	186 (33.0)	<0.001
Prasugrel, *n* (%)	4 (0.3)	1 (0.2)	0.976

*ASA* acetylsalicylic acid, *VKA* vitamin K antagonists, *ACEI* angiotensin-converting-enzyme inhibitor, *ARB* angiotensin receptor blocker, *Ca-blockers* calcium channel blockers

### Interventional treatment and reperfusion strategy

There was a trend for a higher SYNTAX score in our elderly patients (15 IQR 8–26 vs. 14 IQR 8–22; *p* = 0.08). As well, our elderly patients had more multivessel diseases. As shown in Table 3, first- and second-generation DES was used with similar frequency in both groups of patients.

**Table 3:** Angiographic and procedural data according to the age

	Age <70 *n* = 1353 (70.6%)	Age ≥70 *n* = 563 (29.4%)	*p*
SYNTAX score, median (IQR)	14 (8–22)	15 (8–26)	0.082
DES-I, *n* (%)	454 (33.6)	191 (33.9)	0.917
DES-II, *n* (%)	899 (66.4)	372 (66.1)	
No. of vessels with significant stenosis, *n* (%)			
1	517 (38.2)	198 (35.2)	0.015
2	493 (36.4)	186 (33.0)	
3	343 (25.4)	179 (31.8)	
Target vessel, *n* (%)			
Left main	79 (5.8)	46 (8.2)	0.157
Left anterior desc.	701 (51.8)	275 (48.8)	
Left circumflex	245 (18.1)	94 (16.7)	
Right coronary artery	266 (19.7)	114 (20.2)	
Arterial bypass graft	9 (0.7)	2 (0.4)	
Saphenous vein graft	53 (3.9)	32 (5.7)	
Extensive calcifications, *n* (%)	111 (8.2)	56 (9.9)	0.200
Glycoprotein IIb/IIIa inhibitors, *n* (%)	75 (5.5)	21 (3.7)	0.123
Stent thrombosis in culprit lesion, *n* (%)	7 (0.5)	1 (0.1)	0.450
Average stent diameter (mm), median (IQR)	3.0 (2.5–3.5)	3.0 (2.7–3.5)	0.729
Total stent length (mm), median (IQR)	23 (15.0–28.2)	22 (15–28)	0.423
Residual stenosis post-PCI, *n* (%)	9 (0.6)	8 (1.4)	0.108
TIMI 3 flow post-PCI, *n* (%)	1341 (99.1)	554 (98.4)	0.173

*DES-I* first-generation drug-eluting stents, *DES-II* second-generation drug-eluting stents, *TIMI* thrombosis in myocardial infarction, *PCI* percutaneous coronary intervention

### In-hospital outcomes

**Fig. 1:**
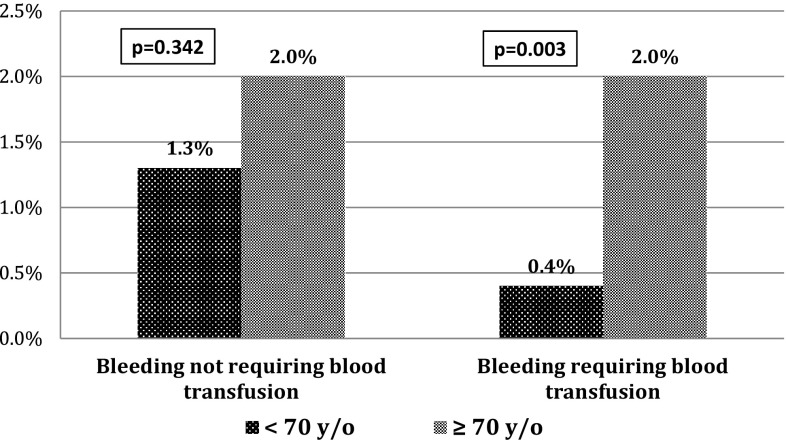
In-hospital bleeding complications according to the age

There was a higher rate of in-hospital bleeding complications requiring blood transfusion in the elderly patients (2.0 vs. 0.9%; *p* = 0.003), compared to the younger aged group (Fig. 1). Even so, there were no differences in rates of acute and subacute ST in both groups of patients. There were more resuscitated cardiac arrests (2.0 vs. 0.9%; *p* = 0.084) in the elderly patients during hospitalization. The composite in-hospital rate of MACCE did not differ statistically between these two groups (1.4 vs. 1.1%; *p* = 0.567) (Table 4).

**Table 4:** In-hospital and 12-month follow-up according to the age

	Age <70 *n* = 1353 (70.6%)	Age ≥70 *n* = 563 (29.4%)	*p*
In-hospital adverse events			
Cardiogenic shock, *n* (%)	12 (0.9)	6 (1.1)	0.912
Respiratory insufficiency, *n* (%)	5 (0.4)	6 (1.1)	0.123
Cardiac arrest, *n* (%)	12 (0.9)	11 (2.0)	0.084
Death, *n* (%)	7 (0.5)	7 (1.2)	0.089
MI, *n* (%)	9 (0.6)	3 (0.5)	0.738
TVR, *n* (%)	9 (0.6)	2 (0.3)	0.413
Stroke, *n* (%)	0 (0)	0 (0)	–
MACCE, *n* (%)	15 (1.1)	8 (1.4)	0.567
12-Month adverse events			
Death, *n* (%)	25 (1.8)	40 (7.1)	<0.001
MI, *n* (%)	66 (4.8)	34 (6.0)	0.300
TVR, *n* (%)	134 (9.9)	41 (7.2)	0.075
Stroke, *n* (%)	9 (0.6)	5 (0.8)	0.600
MACCE, *n* (%)	182 (13.4)	85 (15.0)	0.324
Stent thrombosis			
Acute, *n* (%)	8 (0.6)	2 (0.4)	0.760
Subacute, *n* (%)	5 (0.4)	2 (0.4)	0.712
Late, *n* (%)	3 (0.2)	2 (0.3)	0.975
Gastrointestinal bleeding events in 12-month follow-up, *n* (%)	13 (1.0)	9 (1.6)	0.338

*MI* myocardial infarction, *TVR* target vessel revascularization, *MACCE* major adverse cardiac and cerebral events

### 12-Month follow-up

**Fig. 2:**
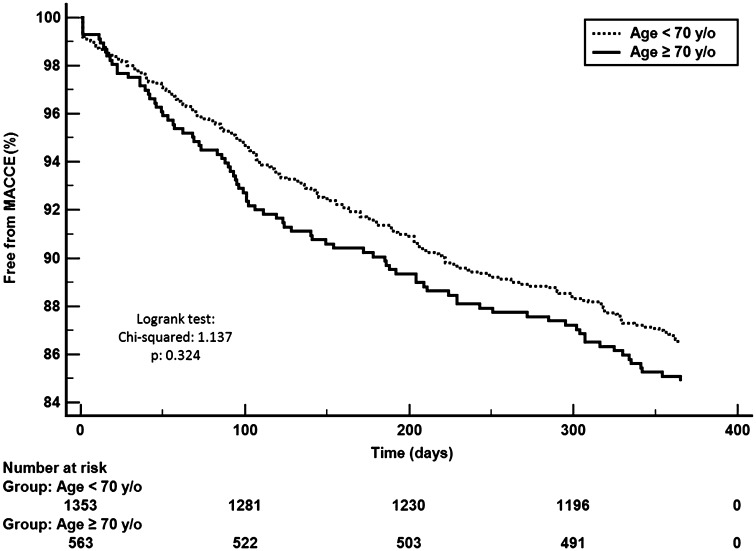
Kaplan–Meier curves for MACCE according to the age

**Fig. 3:**
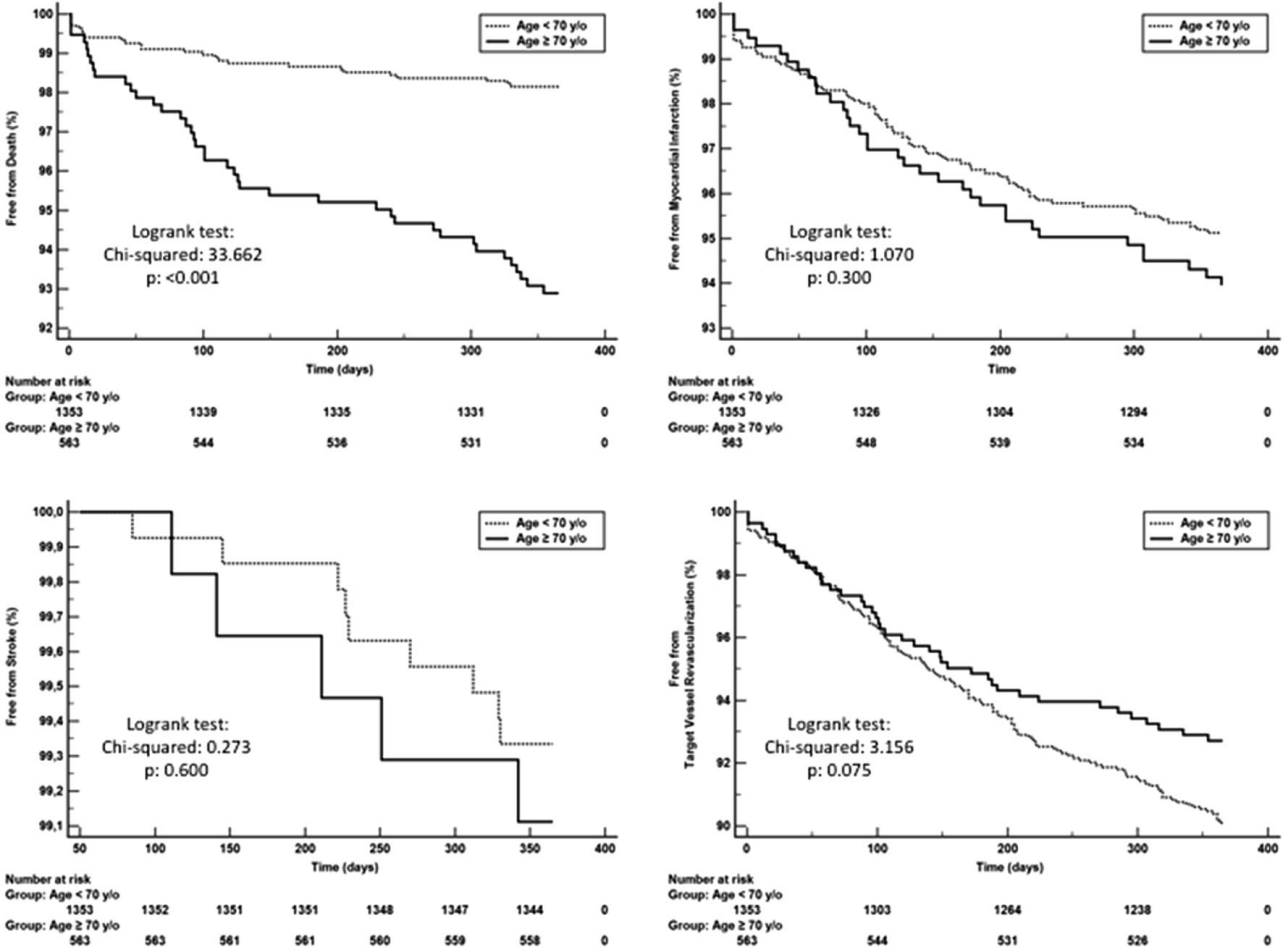
Kaplan–Meier curves for death, myocardial infarction, stroke, TVR according to the age

Although a higher all-cause mortality rate was noted in the elderly patients (7.1 vs. 1.8%, *p* < 0.001), there were no differences in the frequency of composite MACCE (15.0 vs. 13.4%, *p* = 0.324) at 12-month follow-up. There also were no differences in the frequency of repeated revascularizations (7.2 vs. 9.9%, *p* = 0.075), MI (6.0 vs. 4.8%, *p* = 0.300) and strokes (0.8 vs. 0.6%, *p* = 0.600). Data used for the 12-month MACCE probability were presented using Kaplan–Meier curves stratified according to age (Figs. 2, 3). Twelve-month cumulative rate of late stent thrombosis did not differ significantly between these two groups of patients (*p* = 0.975). The rate of 12-month follow-up gastrointestinal bleeding was low and did not differ between groups (*p* = 0.338) (Table 4). The multivariable Cox regressions analysis revealed that age ≥70 was the independent predictor of death [HR = 2.55 (95% CI 1.49–4.37); *p* < 0.001] at 12-month follow-up (Table 5).

**Table 5:** Univariable and multivariable Cox regression analysis

	Predictors of death					
	Univariable analysis			Multivariable analysis		
Age ≥70 y/o	1.07	1.04–1.10	<0.001	2.55	1.49–4.37	<0.001
CKD	2.72	2.28–6.06	<0.001	2.11	1.23–3.60	<0.001
DM	1.98	1.21–3.21	<0.001	1.56	0.95–2.55	0.070
Anemia	2.60	1.48–4.56	<0.001	1.59	0.89–2.86	0.116
Bleeding with blood transfusion	10.38	4.19–25.75	<0.001	4.35	1.68–11.28	0.038
Neoplasm	2.20	1.05–4.59	0.036	1.65	0.78–3.84	0.187

CKD was defined as estimated GFR (eGFR) <60 60 ml/min/1.73 m^2^ calculated using the modification of diet in renal disease (MDRD) method

*CKD* chronic kidney disease, *DM* diabetes mellitus

### Comparison of first- and second-generation DES in elderly patients

**Fig. 4:**
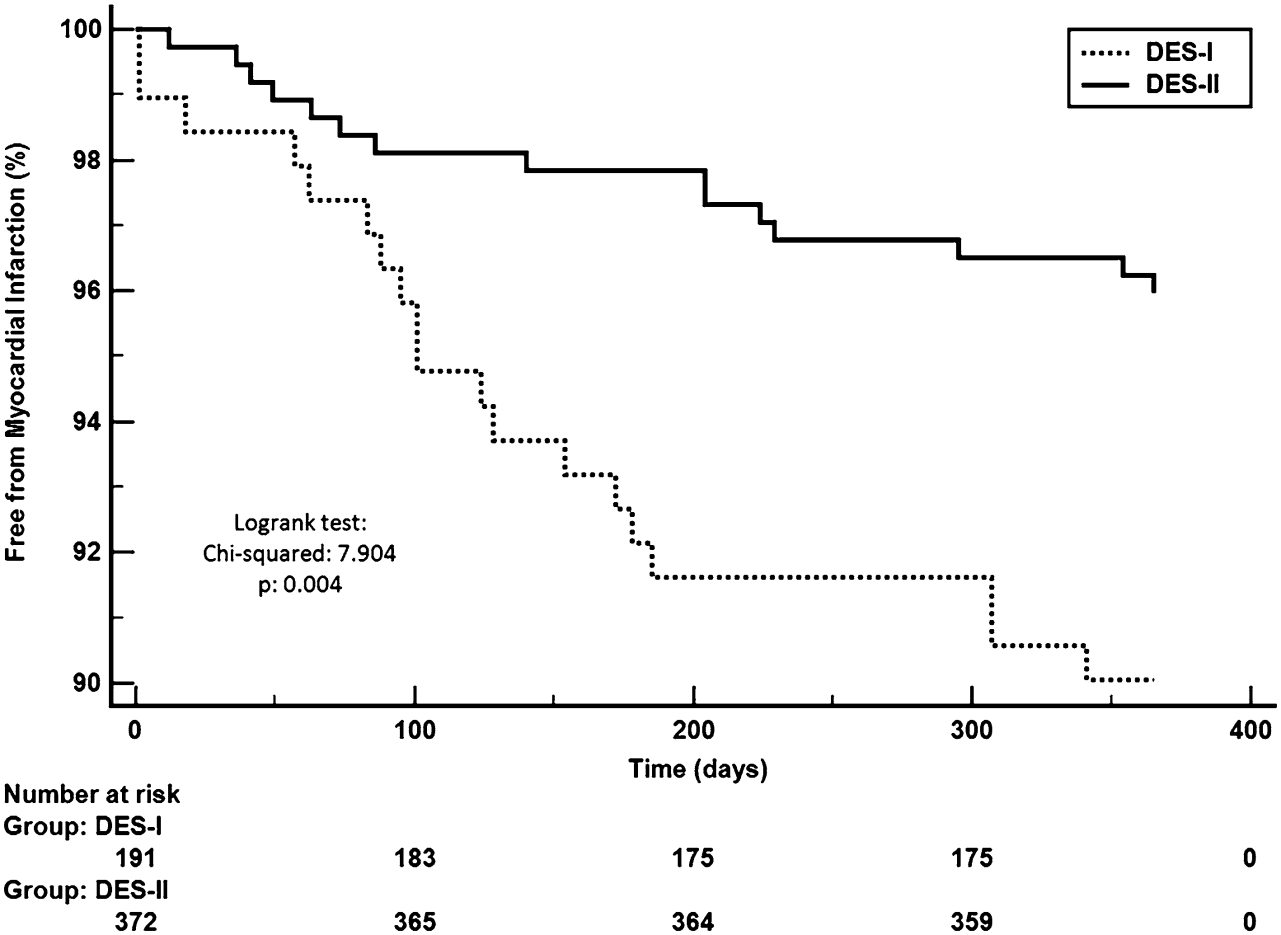
Kaplan–Meier curves for myocardial infarction in patients ≥70 y/o (DES-I vs. DES-II)

There was a lower incidence of MI in the elderly patients treated with DES-II as compared to DES-I (6.7 vs. 9.9%, *p* = 0.004), without significant differences in rate of death (7.5 vs. 6.2%, *p* = 0.586), TVR (5.9 vs. 9.9%, *p* = 0.081), stroke (0.5 vs. 1.5%, *p* = 0.216) and MACCE (13.1 vs. 18.8%, *p* = 0.075) at 12-month follow-up. The use of DES-II reduced the risk of MI [HR = 0.40 (95% CI 0.19 - 0.82); *p* = 0.012] in the elderly. MI probability was presented using Kaplan–Meier curves and stratified according to DES generation (Fig. 4).

## Discussion

We were able to observe during this study that a high proportion of patients hospitalized with ACS were age 70 or over. ACS is known as an important risk factor for cardiovascular events. The main results from our current analysis obtained from Katowice–Zabrze registry’ data are that elderly patients have higher rates of death in a one-year follow-up study, more bleeding complications post-PCI, requiring blood transfusions despite the fact that they do not have a higher risk of acute, subacute and late stent thrombosis. The presence of both risk factors (age and acute presentation) identified a cohort of patients with a high risk of complications after PCI [4, 11, 12]. The current study demonstrated some significant differences between patients representing these two age ranges. Similarly to results observed in other analysis, elderly patients from our registry carry a higher risk of adverse events. They have a higher percentage of comorbidities: hypertension, diabetes, chronic kidney disease, chronic obstructive pulmonary disease, heart failure and higher GRACE risk score as compared to younger group [13, 14]. Our elderly group did not manifest typical angina as would be expected since time to reperfusion in ACS is longer [15, 16]. Analysis of available angiographic data demonstrated that this population of elderly patients differed regarding the complex coronary atherosclerotic lesions when compared to our younger patient groups. As observed in a study by Rosengren et al. [14], these patients have more often multivessel disease and more complex PCI such as left main or saphenous vein graft interventions. Moreover, there were also differences in the efficacy of PCI. Dziewierz et al. [5] analyzed 1650 patients with STEMI and analyzed the outcomes in age strata (<65, 65–74, 75–84 and ≥85 years). They demonstrated that elderly patients were less likely to achieve TIMI 3 flow and ST-segment resolution after PCI and were more likely to have PCI complications.

It is well known that elderly patients with CAD have a worse prognosis when compared with younger patients [5, 14]. Moreover, age is an independent predictor of death and MACCE post-PCI [17, 18]. In this current study, we observed that at 12-month follow-up, rates of death were significantly higher in our elderly patients. However, there were no differences in MACCE. We also observed that elderly patients had more often bleeding complications required a blood transfusion after PCI compared to their younger counterparts. Bleeding is the most frequent non-ischemic complication observed in ACS patients [19]. In addition to such factors as male sex, chronic kidney disease and anemia, older age also increases the risk of bleeding complications in ACS [20–23]. Therefore, these patients need to have individualized antiplatelet therapy to decrease thrombotic events without increasing bleeding [24, 25]. Additionally, choosing the best vascular approach during PCI can significantly reduce the risk of bleeding. Radial access is associated with significant reduction in major bleeding and need for blood transfusions [26].

Stent technology has progressed from bare-metal stents (BMS) to first- and second-generation DES. Data from SCAAR registry indicated that PCI with DES-II was associated with lower risk of clinically significant restenosis, stent thrombosis, and a lower risk of death compared with DES-I in the real-world population [27]. Limited information is available which compared two types of generational DES used in elderly patients. There were studies, which compared DES with bare-metal stents (BMS) in elderly. de Belder et al. [28] published data from a randomized multicenter trial, comparing everolimus-eluting stents (EES) with BMS in octogenarian patients with stable angina and ACS. He reported that there were no differences of all-cause death, stroke and bleeding complications. However, use of EES reduced the incidence of MI and TVR in a 1-year follow-up. In our present study, we compared data from DES-I with DES-II used in elderly patients. DES-II was associated with reduced incidence of MI in the following year, but there were no differences in the incidence of death, TVR, stroke or MACCE. Our results confirmed previous observations and, as suggested in ESC guidelines, advocated the use of DES-II for PCI regardless of patient’s characteristic [29].

## Study limitations

Patients were not randomized as to a choice of stent implantation (DES first or second generation), so there was no balance between DES-I and DES-II. There was no information on drugs used before admission to the hospital, especially those with a known impact on the occurrence of bleeding. There was no information about the duration of medication (e.g., patients taking clopidogrel, prasugrel) after PCI.

## Conclusion

Elderly patients had an increased risk of in-hospital bleeding requiring blood transfusion and had a higher risk of death at 12-month follow-up. The use of the new generation of DES reduced the risk of MI in the elderly at 12-month follow-up.
